# Sociodemographic predictive factors of increased hospital stay and cost among hospitalised patients with pressure injuries—National Inpatient Sample 2009–2019 (pooled sample)

**DOI:** 10.1111/wrr.70027

**Published:** 2025-04-22

**Authors:** Siri Choragudi, Robert S. Kirsner

**Affiliations:** ^1^ Dr. Philip Frost Department of Dermatology and Cutaneous Surgery University of Miami Miller School of Medicine Miami Florida USA; ^2^ Transitional Year Residency Program Baylor Scott & White All Saints Medical Center Fort Worth Texas USA

**Keywords:** healthcare disparities, hospital costs, National Inpatient Sample, pressure injuries, sociodemographic factors

## Abstract

Pressure injuries (PIs) pose a significant health burden, particularly among hospitalised patients. Understanding the sociodemographic factors influencing length and cost of hospital stay is crucial for addressing healthcare disparities. This study investigated the associations between sociodemographic factors and hospital stay outcomes among patients with pressure injuries in the United States from 2009 to 2019. Using the National Inpatient Sample (NIS), we analysed data from 1,252,729 patients with pressure injuries. We performed survey‐weighted multivariable linear regression to assess the impact of sociodemographic factors on hospital stay duration and costs. Minority racial groups, lower‐income patients, and those with non‐Medicare insurance experienced longer hospital stays and higher costs. Older age groups (≥30 years) had shorter stays and lower costs. Significant disparities exist in hospital outcomes for patients with pressure injuries. Targeted interventions are needed to address these inequalities and improve patient care.

## 1 INTRODUCTION

Pressure injuries (PIs) are a prevalent and serious health concern, affecting up to 3 million individuals annually in the United States.^1^, ^2^ These injuries result from prolonged tissue ischemia due to sustained pressure and are particularly common in hospitalised patients, especially those in intensive care units or with comorbid conditions such as spinal cord injuries, malnutrition or diabetes mellitus.^3^ PIs not only lead to significant patient morbidity but also result in increased healthcare costs and extended hospital stays.^4^ Despite extensive research on the clinical management of PIs, the influence of sociodemographic factors on hospital stay duration and costs remains underexplored.

Several studies have suggested that sociodemographic factors, such as race and socioeconomic status, may contribute to disparities in healthcare outcomes—including the incidence and management of PIs.^5^, ^6^ However, no comprehensive analysis has been conducted to specifically examine how these factors influence the length of hospital stay and associated costs for patients with PIs in the United States. Understanding these associations is crucial for addressing healthcare disparities and improving outcomes for vulnerable populations.

## 2 METHODS

### 2.1 Study design and data source

We conducted a retrospective cohort study using data from the National Inpatient Sample (NIS), a nationwide database maintained by the Healthcare Cost and Utilisation Project (HCUP). The NIS represents 20% of hospitalizations in the United States and enables national‐level estimates.^7^ This study included patients aged 18 years or older with a primary or secondary diagnosis of PIs, identified using ICD‐9‐CM codes (707.0, 707.2) for 2009–2015 and ICD‐10‐CM codes (L89) for 2016–2019.^8^

### 2.2 Variables

The primary outcomes were the length of hospital stay (days) and total hospital costs (in US dollars). The exposure variables included age, gender, race, household income, and insurance type. Race was categorised as White, Black, Hispanic, and Other, and income was stratified into four categories (<$39,000; $39,000–$47,999; $48,000–$62,999; ≥$63,000).

### 2.3 Statistical analysis

Descriptive analyses were performed using survey‐weighted means for continuous variables and frequencies for categorical variables. Multivariable linear regression was employed to assess the associations between sociodemographic factors and the outcomes, adjusting for potential confounders.^9^ All analyses were conducted using IBM SPSS® Statistics 25.0, with a significance threshold of *p* < 0.05. This modelling approach allowed us to determine the unique impact of each variable on the outcomes while controlling for the others.

## 3 RESULTS

### 3.1 Sample characteristics

The study analysed 1,252,729 unweighted hospital admissions involving patients diagnosed with PIs between 2009 and 2019. After applying survey weights, this sample corresponded to an estimated 6,252,083 hospitalizations nationwide. The mean age of patients was 70.88 years (SE = 0.06), with a slight majority being women (50.5%). The racial/ethnic distribution indicated that 67.4% of patients were White, 18.1% were Black, 10.3% were Hispanic, and 4.2% were categorised as Other. Most patients (77%) had Medicare as their primary insurance, while 32.4% reported an annual household income of less than $39,000.

The analysis identified several sociodemographic factors significantly associated with higher inpatient burden—defined as longer hospital stays and higher costs—among patients with PIs. Notably, patients over 90 years of age had the shortest hospital stay duration, remaining 4.01 days less than the reference group (aged 18–29 years) (*p* < 0.01). This age group also incurred the most substantial decrease in hospital costs, with $51,877.35 less compared to the reference group (*p* < 0.01) (Table 1).

**TABLE 1:** Associations of sociodemographic factors with length and cost of hospital stay among hospitalised patients with pressure injuries—National Inpatient Sample 2009–2019 (pooled sample).

Demographic characteristics	Length of stay (days)			Total cost ($)		
	Mean (SE)	Mean difference (SE)	*p*‐value	Mean (SE)	Mean difference (SE)	*p*‐value
Age categories						
18–29 years	14.08 (0.17)	Ref.	Ref.	132748.18 (2155.30)	Ref.	Ref.
30–39 years	13.29 (0.13)	**−0.78** (0.18)	<0.01	121909.10 (1573.91)	**−10839.08** (2186.97)	<0.01
40–49 years	13.79 (0.12)	−0.28 (0.16)	0.09	124834.06 (1330.53)	**−7914.11** (2022.98)	<0.01
50–59 years	13.81 (0.11)	−0.26 (0.16)	0.11	125662.34 (1136.06)	**−7085.83** (1939.34)	<0.01
60–69 years	13.56 (0.10)	**−0.52** (0.16)	<0.01	124925.43 (1082.73)	**−7822.74** (1956.63)	<0.01
70–79 years	12.73 (0.10)	**−1.35** (0.16)	<0.01	112416.87 (999.28)	**−20331.30** (1952.29)	<0.01
80–89 years	11.44 (0.09)	**−2.64** (0.16)	<0.01	94757.82 (920.01)	**−37990.36** (1973.65)	<0.01
90 years or more	10.07 (0.09)	**−4.01** (0.17)	<0.01	80870.82 (900.70)	**−51877.35** (1990.36)	<0.01
Sex						
Male	13.16 (0.09)	Ref.	Ref.	118963.92 (1044.74)	Ref.	Ref.
Female	12.53 (0.09)	**−0.63** (0.03)	<0.01	110567.24 (994.18)	**−8396.67** (294.30)	<0.01
Race						
White	11.64 (0.07)	Ref.	Ref.	94678.74 (859.87)	Ref.	Ref.
Black	12.80 (0.09)	**+1.17** (0.06)	<0.01	102899.11 (1061.60)	**+8220.37** (726.33)	<0.01
Hispanic	13.06 (0.13)	**+1.42** (0.11)	<0.01	132325.31 (1483.82)	**+37646.57** (1284.57)	<0.01
Other	13.89 (0.18)	**+2.25** (0.16)	<0.01	129159.15 (1686.60)	**+34480.42** (1443.21)	<0.01
Income						
<$39,000	12.69 (0.10)	Ref.	Ref.	106,797.56 (1012.68)	Ref.	Ref.
$39,000–$47,999	12.70 (0.09)	0.01 (0.05)	0.93	110,544.65 (1020.47)	**+3747.08** (567.45)	<0.01
$48,000–$62,999	12.78 (0.10)	0.09 (0.06)	0.14	116,377.31 (1085.24)	**+9579.74** (694.26)	<0.01
$63,000 or more	13.22 (0.11)	**+0.53** (0.08)	<0.01	125,342.80 (1349.50)	**+18,545.24** (1047.02)	<0.01
Insurance type						
Medicare	10.79 (0.07)	Ref.	Ref.	99,506.83 (811.15)	Ref.	Ref.
Medicaid	13.79 (0.13)	**+3.00** (0.13)	<0.01	122,096.86 (1190.78)	**+22,590.03** (920.12)	<0.01
Private or HMO	12.81 (0.10)	**+2.01** (0.07)	<0.01	122,209.48 (1285.28)	**+22,702.65** (853.92)	<0.01
Self‐pay	14.71 (0.22)	**+3.91** (0.21)	<0.01	120,722.85 (2252.66)	**+21,216.02** (2184.89)	<0.01
Other	12.13 (0.16)	**+1.33** (0.15)	<0.01	109,291.88 (1755.06)	**+9785.05** (1564.38)	<0.01

*Note:* Statistically significant differences with *p* < 0.05 in multivariable regression analysis are mentioned in bold.

Hispanic patients experienced the highest increase in hospital costs, incurring an additional $37,646.57 compared to White patients (*p* < 0.01). They also had a significantly longer hospital stay, averaging 1.42 days more than White patients (*p* < 0.01). Similarly, patients identified as ‘Other Race’ had the longest increase in hospital stay duration—2.25 days more than White patients—and incurred an additional $34,480.42 in hospital costs (*p* < 0.01) (Table 1).

Black patients also experienced a significant increase in hospital stay duration, staying 1.17 days longer than White patients (*p* < 0.01), and incurred $8220.37 more in costs compared to White patients (*p* < 0.01). In contrast, female patients had shorter stays by 0.63 days compared to males (*p* < 0.01) and incurred $8396.67 less in hospital costs (*p* < 0.01) (Table 1).

Self‐pay patients had the longest hospital stays, with an increase of 3.91 days compared to Medicare patients (*p* < 0.01), and faced significantly higher costs, incurring an additional $21,216.02 compared to Medicare patients (*p* < 0.01). Additionally, patients with an income of ≥$63,000 experienced slightly longer stays (an increase of 0.53 days) and incurred $18,545.24 more in costs compared to those with incomes below $39,000 (*p* < 0.01) (Table 1).

## 4 DISCUSSION

This study utilised a large, nationally representative sample to analyse the impact of sociodemographic factors on the length and cost of hospital stays among inpatients with PIs in the United States from 2009 to 2019. The results highlighted significant disparities in healthcare outcomes associated with age, gender, race, income, and insurance type, with minority racial groups and those with non‐Medicare insurance experiencing longer hospital stays and higher costs.^2^

The most significant predictive factors for higher inpatient burden were associated with race, particularly among Hispanic and ‘Other Race’ patients, followed by insurance type and income level. Age also played a significant role, particularly among the elderly, who tended to have shorter stays and lower costs. These findings align with previous studies that have identified racial and socioeconomic disparities in healthcare outcomes, particularly regarding prolonged hospitalizations and increased healthcare costs among minority patients.^10^

Hispanic patients, for instance, had a significantly longer hospital stay by 1.42 days and incurred $37,646.57 more in costs compared to White patients (Table 1). Similarly, patients identified as ‘Other Race’ had the longest increase in hospital stay duration by 2.25 days and the highest associated cost increase of $34,480.42 compared to White patients (Table 1). These findings underscore the systemic inequalities that persist in healthcare, where minority groups often face greater barriers to receiving timely and effective care.^11^ These disparities may be attributed to a variety of factors, including differences in healthcare access, socioeconomic status, health literacy, and potential biases within the healthcare system.^12^

The significant burden observed among self‐pay patients, who had the longest hospital stays (an increase of 3.91 days) and incurred substantially higher costs ($21,216.02 more) compared to Medicare patients (Table 1), highlights the financial vulnerabilities of those without Medicare or private insurance coverage. These patients may encounter barriers to timely medical intervention, which could contribute to extended hospital stays and increased healthcare costs.^13^ This finding underscores the importance of policies aimed at improving healthcare access and financial assistance programs to support patients across different insurance categories.^14^, ^16^

Income level was another critical factor influencing hospital outcomes. Patients with higher incomes (≥$63,000) experienced slightly longer stays (0.53 days) and incurred higher costs ($18,545.24) compared to those with lower incomes (Table 1). While this might seem counterintuitive, it could reflect differences in care‐seeking behaviour, with higher‐income patients potentially undergoing more comprehensive or elective treatments. Alternatively, this disparity might be related to variations in hospital resource utilisation or differences in the types of facilities accessed by higher‐income individuals. Further research is needed to clarify these associations and determine the underlying mechanisms driving these differences.^15^

The role of age in determining inpatient burden was also evident in our analysis.^16^ Interestingly, patients aged 90 years or more had the shortest hospital stays—with a reduction of 4.01 days—and the lowest costs, saving $51,877.35 compared to the reference group aged 18–29 years (Table 1). This finding may suggest that the shorter length of stay observed among the elderly could be, in part, due to higher in‐hospital mortality, given that the NIS dataset does not distinguish between recovery and discharge due to death. Conversely, younger patients (aged 18–29 years) had the longest stays and highest costs, possibly reflecting more complex or acute conditions requiring extended treatment.^17^ These findings highlight the need for age‐specific approaches to managing pressure injuries, particularly for younger patients who may require more intensive care and longer recovery periods.

Despite the strengths of this study, including its large sample size and comprehensive analysis of multiple sociodemographic factors, there are limitations to consider. The use of hospital cost and length of stay as primary measures of PI burden may provide a narrow interpretation of the overall impact on patients. Other factors, such as quality of life, functional status and long‐term care needs, should be explored in future research to provide a more holistic understanding of the burden of pressure injuries. Additionally, while we controlled for several sociodemographic variables, residual confounding due to unmeasured factors (e.g., access to healthcare, patient comorbidities and variations in provider practices) may have influenced our results.^18^

## 5 CONCLUSION

This study highlights significant healthcare disparities in the management of pressure injuries, particularly among minority racial groups, uninsured or self‐pay patients, and those with lower socioeconomic status. Race emerged as a key predictor, with Hispanic and ‘Other Race’ patients experiencing the most substantial increases in both hospital stay duration and associated costs. These findings underscore the urgent need for culturally sensitive interventions and policies aimed at reducing racial inequalities in healthcare.

Additionally, the financial vulnerability of self‐pay patients, who faced the longest stays and highest costs, emphasises the importance of expanding insurance coverage and implementing financial assistance programmes. The study also suggests that income level may influence healthcare utilisation patterns, with higher‐income patients experiencing longer stays and higher costs.

Age‐specific care needs were evident, with elderly patients benefiting from more streamlined care, while younger patients incurred a higher inpatient burden—indicating the need for tailored strategies. Moving forward, targeted interventions are essential to address these disparities, improve access to care and ensure equitable treatment across different sociodemographic groups.

Future research should expand on these findings by exploring outcomes beyond hospital stay and cost—including quality of life and long‐term health impacts—to provide a more comprehensive understanding of the burden of pressure injuries and the measures needed to address it effectively.

## CONFLICT OF INTEREST STATEMENT

The authors declare that they have no conflict of interest.
